# Investigation of Impact of Oxidative Stress on Human Periodontal Ligament Cells Exposed to Static Compression

**DOI:** 10.3390/ijms252413513

**Published:** 2024-12-17

**Authors:** Samira Hosseini, Julia Diegelmann, Matthias Folwaczny, Iris Frasheri, Andrea Wichelhaus, Hisham Sabbagh, Corrina Seidel, Uwe Baumert, Mila Janjic Rankovic

**Affiliations:** 1 Department of Orthodontics and Dentofacial Orthopedics, LMU University Hospital, LMU Munich, 80336 Munich, Germany; samirahosseini1251@gmail.com (S.H.); kfo.sekretariat@med.uni-muenchen.de (A.W.); corinna.seidel@med.uni-muenchen.de (C.S.); uwe.baumert@med.uni-muenchen.de (U.B.); 2 Department of Conservative Dentistry and Periodontology, LMU University Hospital, LMU Munich, 80336 Munich, Germany; julia.diegelmann@med.uni-muenchen.de (J.D.); matthias.folwaczny@med.uni-muenchen.de (M.F.); iris.frasheri@med.uni-muenchen.de (I.F.)

**Keywords:** oxidative stress, human periodontal ligament cells, static compressive force, bone remodeling, orthodontic tooth movement

## Abstract

Oxidative stress (OS) is a common feature of many inflammatory diseases, oral pathologies, and aging processes. The impact of OS on periodontal ligament cells (PDLCs) in relation to oral pathologies, including periodontal diseases, has been investigated in different studies. However, its impact on orthodontic tooth movement (OTM) remains poorly understood. This study used an in vitro model with human PDLCs previously exposed to H_2_O_2_ to investigate the effects of OS under a static compressive force which simulated the conditions of OTM. Human PDLCs were treated with varying concentrations of H_2_O_2_ to identify sub-lethal doses that affected viability minimally. To mimic compromised conditions resembling OTM under OS, the cells were pretreated with the selected H_2_O_2_ concentrations for 24 h. Using an in vitro loading model, a static compressive force (2 g/cm^2^) was applied for an additional 24 h. The cell viability, proliferation, and cytotoxicity were evaluated using live/dead and resazurin assays. Apoptosis induction was assessed based on caspase-3/7 activity. The gene expression related to bone remodeling (*RUNX2*, *TNFRSF11B*/*OPG*, *BGLAP*), inflammation (*IL6*, *CXCL8*/*IL8*, *PTGS2*/*COX2*), apoptosis (*CASP3*, *CASP8*), and autophagy (*MAP1LC3A*/*LC3*, *BECN1*) was analyzed using RT-qPCR. This study suggests an altering effect of previous OS exposure on static-compression-related mechanosensing. Further research is needed to fully elucidate these mechanisms.

## 1. Introduction

Oxidative stress (OS) arises from an imbalance between the production of reactive oxygen species (ROS) and the host’s ability to detoxify them or repair the resulting damage [1,2]. In recent years, research has emphasized the role of OS in various oral pathologies, particularly chronic inflammatory conditions like periodontal disease, as well as in physiological processes such as aging [3,4,5,6,7].

The inflammatory response to the presence of periodontal pathogens increases the production of ROS [7,8,9]. Various studies have confirmed a positive correlation between elevated ROS levels and the degree of periodontal tissue inflammation [10]. Excessive ROS accumulation causes OS, leading to the damage of cells and tooth-supporting tissues. This damage triggers the release of more inflammatory mediators, creating a continually reinforcing feedback loop of inflammation and OS [7,9]. This cycle negatively impacts periodontal ligament cells (PDLCs), a dominant cell type in the periodontal ligament (PDL), leading to their dysfunction, apoptosis, and reduced regenerative capacity [11,12]. This ongoing damage and inflammation can result in the destruction of periodontal tissues and impair bone turnover, thus disrupting the balance between bone formation and resorption [13,14]. Additionally, this process negatively affects cellular functions such as autophagy and apoptosis [11,15]. These findings suggest that ROS accumulation might contribute to imbalances in bone metabolism and could adversely affect orthodontic tooth movement (OTM) and post-treatment stability [14].

PDLCs are also known for their crucial role during OTM, where mechanical forces are applied to the teeth to correct their alignment. These forces lead to biological responses in the surrounding tissues, resulting in tooth movement. Elevated pro-inflammatory responses of PDLCs may occur as a consequence of this mechanical stimulation [16]. Particularly, the compression side during OTM is characterized by the presence of sterile inflammation and the increased expression of inflammatory mediators by the PDLCs [16,17]. However, with the exception of few studies that have confirmed increased ROS generation on the compression side during OTM [18,19], the impact of OS and ROS accumulation on OTM at the molecular level is still largely unknown. While lower concentrations of ROS can stimulate tissue-related processes and support regeneration, excessive ROS accumulation—e.g., in chronic inflammatory disorders or with aging-related decline in antioxidant defense—is linked to tissue-destructive processes [3,20,21,22].

Given the growing elderly population and the increasing number of adult orthodontic patients with underlying degenerative inflammatory conditions, the goal of our study was to investigate whether pre-exposure to OS alters the cellular response and how this may influence static-compression-related mechanosensing in hPDLCs. For these purposes, we used a combination of two well-established setups: one for OS simulation and the other for OTM simulation. Hydrogen peroxide (H_2_O_2_), a key mediator of OS, has been widely used in experimental settings to simulate OS conditions in cell cultures [11,20,23,24,25,26,27,28]. By introducing controlled levels of H_2_O_2_, the cellular environment observed in aging or disease states can be simulated. On the other hand, in vitro models for the application of mechanical stimulation have been well established in the field of orthodontics for investigating molecular events related to mechanosensing [29]. They allow for an in-depth analysis of the complex situation in vivo and investigation of the molecular biology of OTM. One of these models is the weight-approach-based (WAB) in vitro model for the application of static compression [17,30] that we used in this study. These two models for OS and OTM allowed us to simplify the in-depth analysis of the complex situation in vivo and investigate the molecular biology of OTM, focusing on just one cell type and one type of force.

In this study, suitable experimental parameters were established based on cell viability and proliferation. OS/WAB-related gene expression regulation was analyzed in genes related to bone remodeling (*RUNX2*, *BGLAP*, *TNFRSF11B*/*OPG*), inflammation (*IL6*, *CXCL8*/*IL8*, *PTGS2*), autophagy *(MAP1LC3A*/*LC3*, *BECN1*), and apoptosis (*CASP3*, *CASP8*).

## 2. Results

Initially, based on the recommendations from the literature, an appropriate H_2_O_2_ concentration range for use in hPDLC culture was tested. Based on cell viability and cytotoxicity assessments, as well as apoptosis marker expression, three concentrations (50 µM, 100 µM, and 200 µM) were selected for use in the experiments.

Afterwards, the effect of ROS stimulation in the hPDLC culture was examined using three selected doses of H_2_O_2_. Two timepoints for gene expression investigation were selected, one directly after H_2_O_2_ incubation (the “direct” phase) and the other after 24 h of recovery in H_2_O_2_-free medium (the “recovery” phase). In this way, it was possible not only to see how different ROS concentrations affected the gene expression related to inflammation, bone remodeling, and tissue homeostasis but also to confirm and examine the consequent long-term changes in cellular behavior.

Finally, we examined the effects of the application of a compression force to the cells after 24 h of recovery from H_2_O_2_ stimulation. The justification for the application of OS stimulation prior to mechanical stimulation is based on the clinical recommendations to start orthodontic treatment during the remission stage of existing chronical inflammatory disorders.

### 2.1. Hydrogen Peroxide (H_2_O_2_) Concentration Testing

**Figure 1:**
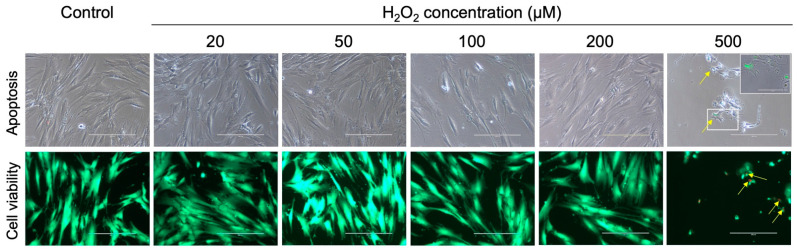
Effect of different hydrogen peroxide concentrations on apoptosis induction (upper row) and cell viability (lower row) in hPDLCs. Upper row: Apoptosis detection using CellEvent™ Caspase-3/7 Detection Reagent (green fluorescence; yellow arrows) in hPDLCs exposed to different H_2_O_2_ concentrations (0 µM to 500 µM). Insert: Area with higher magnification shows green fluorescence. Lower row: Live/dead cell staining of hPDLCs treated with the different H_2_O_2_ concentrations. Green cells indicate viability, whereas dead cells are either detached or stained red (yellow arrows). Fluorescence microscopy was carried out using an EVOS^®^*fl* microscope (Invitrogen, Carlsbad, CA, USA) (Scale bar: 200 µm).

Firstly, as part of establishing an OS model, a range of H_2_O_2_ concentrations for use in the hPDLC culture was evaluated based on the recommendations from the literature [11,20,24]. To determine the optimal concentrations of H_2_O_2_ that induce cytotoxic effects without compromising the cell viability, the hPDLCs were exposed to rising concentrations of H_2_O_2_ from 0 µM to 500 µM. Following an assessment of apoptosis and cell viability, concentrations of 50 µM, 100 µM, and 200 µM were identified as suitable for further experimental steps (Figure 1).

**Figure 2:**
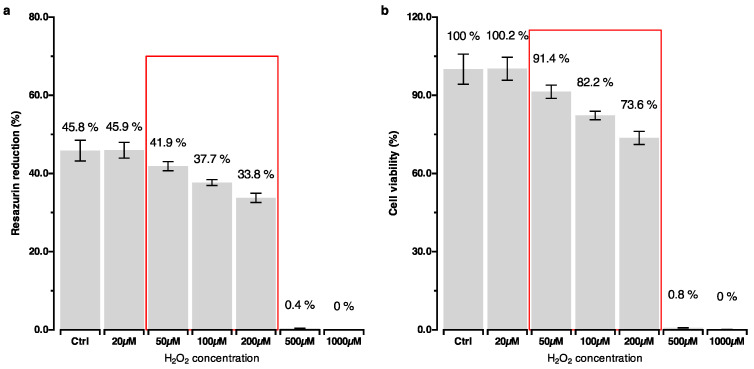
Percentage reduction in resazurin. (**a**) Cytotoxic effect of H_2_O_2_; (**b**) cell viability calculated as normalized resazurin reduction relative to that in the control group. 50 µM, 100 µM, and 200 µM were identified as the lowest concentrations of H_2_O_2_ that showed a cytotoxic effect, however, without a pronounced effect on cell viability.

The results from the resazurin test, used to quantitatively assess cytotoxicity and cell viability, supported the selection of 50 µM, 100 µM, and 200 µM of H_2_O_2_ as minimally cytotoxic while retaining cellular viability (Figure 2). These findings were in line with the qualitative results from the microscopic imaging.

### 2.2. Investigation of H_2_O_2_′s Effect on Gene Expression Directly After Incubation and 24 h Post-Incubation

**Figure 3:**
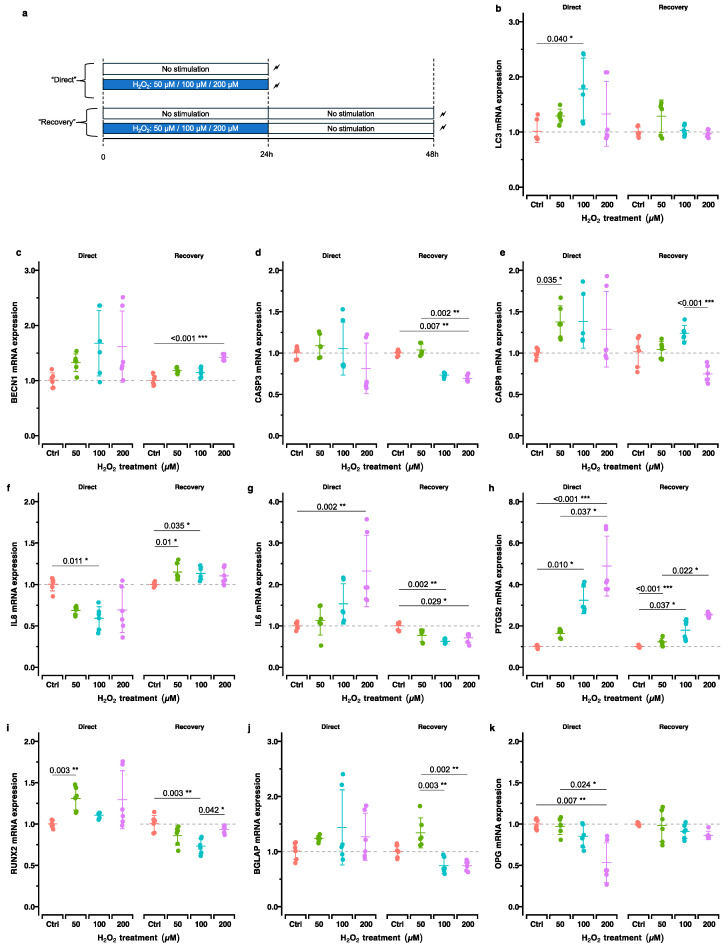
Effect of OS induction alone on gene expression immediately after H_2_O_2_ incubation (“direct”) and 24 h post-incubation (“recovery”). (**a**) Experimental design. (**b**–**k**) RT-qPCR results for genes related to autophagy ((**b**,**c**) *MAP1LC3A*/*LC3*, *BECN1*), apoptosis ((**d**,**e**), *CASP3*, *CASP8*), inflammation ((**f**,**h**), *CXCL2*/*IL8*, *IL6*, *PTGS2*/*COX2*), and bone remodeling ((**i**,**k**), *RUNX2*, *P2RX7*, *TNFRSF11B*/*OPG*). For each genetic locus, the gene expression directly after H_2_O_2_ exposure (left panel, “direct”) and after an additional 24 h of cultivation in H_2_O_2_-free cell culture medium (right panel, “recovery”) is depicted. Adjusted *p*-values (*p*_adj_.) based on multiple comparisons within each group are reported: *, *p*_adj_. < 0.05; **, *p*_adj_. < 0.01; ***, *p*_adj_. < 0.001.

To assess the effect of OS on the hPDLCs’ gene expression profile, we focused on the impact of H_2_O_2_ (50 µM, 100 µM, and 200 µM) on the cells directly after its application (“direct”) and 24 h post-incubation (“recovery”). The expression of genes related to bone remodeling, inflammation, autophagy and apoptosis was assessed using RT-qPCR (Figure 3).

The autophagy-related genes *MAP1LC3A*/*LC3* and *BECN1* showed an overall upregulation which was statistically significant, directly after stimulation with 100 μM of H_2_O_2_ for *MAP1LC3A*/*LC3* and after recovery from 200 µM of H_2_O_2_ for *BECN1* (Figure 3b,c). The apoptosis-related genes caspase-3 (*CASP3*) and caspase-8 (*CASP8*) showed no significant changes in expression across the H_2_O_2_-stimulated groups, except for a decrease in *CASP3* expression after recovery from the treatment with 200 μM H_2_O_2_ and an increase in *CASP8* expression immediately following stimulation with 50 μM H_2_O_2_ (Figure 3d,e).

Our results showed a complex time- and H_2_O_2_-dose-dependent regulation pattern of the key inflammatory mediators *PTGS2*/*COX2*, *CXCL8*/*IL8*, and *IL6* in response to H_2_O_2_ stimulation (Figure 3f–h). *CXCL8*/*IL8* showed a biphasic response: initial downregulation immediately after H_2_O_2_ exposure, followed by upregulation after the 24 h recovery period (Figure 3f). Interestingly, IL6 had the opposite pattern: immediate upregulation after H_2_O_2_ exposure, followed by downregulation after recovery (Figure 3g). *PTGS2*/*COX2* displayed consistent upregulation with increasing H_2_O_2_ concentrations both in the “direct” and “recovery” groups in a dose-dependent manner (Figure 3h).

Overall, the bone-remodeling-related genes exhibited a stable expression pattern, characterized by minimal up- or downregulation in different conditions (Figure 3i–k). *RUNX2* showed a significant increase directly after stimulation with 50 μM H_2_O_2_ and furthermore a decrease after recovery from 100 μM H_2_O_2_ (Figure 3i). *BGLAP* demonstrated a significant decrease in expression after recovery from 100 and 200 μM H_2_O_2_ stimulation compared to that with 50 μM H_2_O_2_ (Figure 3j). *TNFRSF11B*/*OPG* was downregulated in the direct phase, with statistical significance observed only at the highest concentration tested (200 μM H_2_O_2_) (Figure 3k).

### 2.3. Expression of Genes and Metabolites Related to Inflammation, Bone Remodeling, Apoptosis, and Autophagy in Compressed Cells With and Without H_2_O_2_ Preincubation

**Figure 4:**
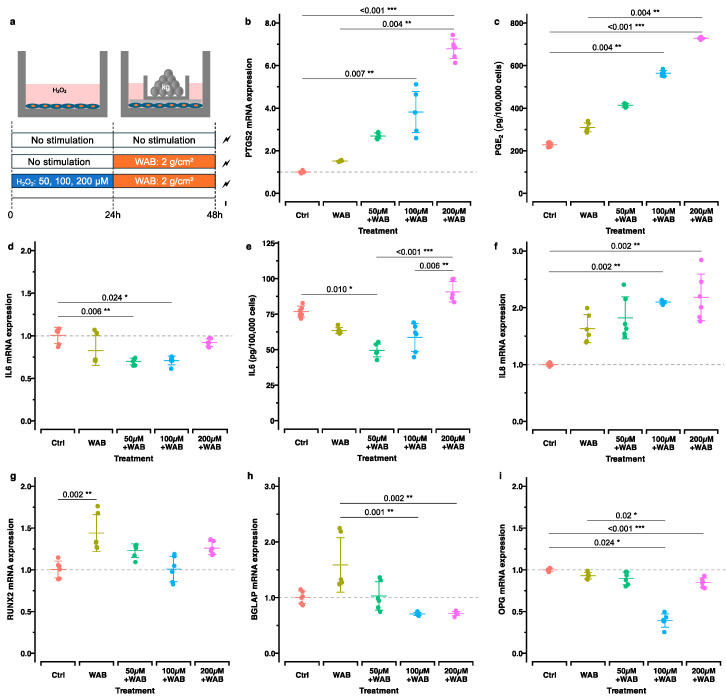
Expression of genes and metabolites related to inflammation, bone remodeling, apoptosis, and autophagy in mechanically stimulated cells with and without previous H_2_O_2_ stimulation. (**a**) Experimental setup: The control group (Ctrl) received neither H_2_O_2_ nor compression stimulation. The compression group (WAB) was stimulated with static compression (2 g/cm^2^) after 24 h of no stimulation. The H_2_O_2_/WAB group was stimulated for 24 h with 50 µM, 100 µM, or 200 µM H_2_O_2_ followed by 24 h of static compression at 2 g/cm^2^. (**b**–**f**) Expression of inflammation-related genes and metabolites and (**g**–**i**) genes related to bone remodeling is reported. Adjusted *p*-values (*p*_adj_.) based on multiple comparisons within each group are reported: *, *p*_adj_. < 0.05; **, *p*_adj_. < 0.01; ***, *p*_adj_. < 0.001.

Next, we examined the effect of the application of H_2_O_2_ in the recovery phase followed by the application of static compression using the “WAB model” [17,30]. The expression patterns of genes and proteins related to inflammation, bone remodeling, apoptosis, and autophagy were analyzed (Figure 4).

#### 2.3.1. Inflammation

Upregulation in the expression of the *PTGS2*/*COX2* gene was observed after the application of static compression in all groups. Especially in the H_2_O_2_-prestimulated groups, a dose-dependent pattern, with the maximal upregulation in the group “200 µM H_2_O_2_ and WAB” (*p*_adj._ < 0.001) (Figure 4b), was found. An almost identical pattern for PGE2 concentration was measured in the corresponding cell culture supernatants (Figure 4c). The compression force also increased *CXCL8*/*IL8* expression (Figure 4f). Like *PTGS2*/*COX2*, the *CXCL8*/*IL8* upregulation was more pronounced in the groups pretreated with H_2_O_2_ in a dose-dependent manner. On the contrary, *IL6* gene expression was generally downregulated after static compression, with or without H_2_O_2_ prestimulation (Figure 4d). The IL6 protein concentration in the cell culture supernatant showed the same pattern except in the group pretreated with 200 μM H_2_O_2_, where upregulation, though not statistically significant, was observed (Figure 4e).

#### 2.3.2. Bone Remodeling

Although not always statistically significantly, all groups of compressed cells previously exposed to H_2_O_2_ showed lower gene expression levels related to osteogenesis in comparison to those in the WAB group where only compression was applied (Figure 4g–i). A significant increase in *RUNX2* gene expression was noted as an effect of static compression in the groups of cells without pretreatment in the OS condition (Figure 4g). In contrast, *TNFRSF11B*/*OPG* showed no significant changes in expression, except after the application of a compression force following recovery from 100 μM H_2_O_2_ stimulation (Figure 4i). The osteogenic gene *BGLAP* showed a significant decrease in expression after applying a compression force to cells preincubated with 100 μM and 200 μM H_2_O_2_ compared to that in the cells that just received static compression (Figure 4h).

#### 2.3.3. Apoptosis and Autophagy

**Figure 5:**
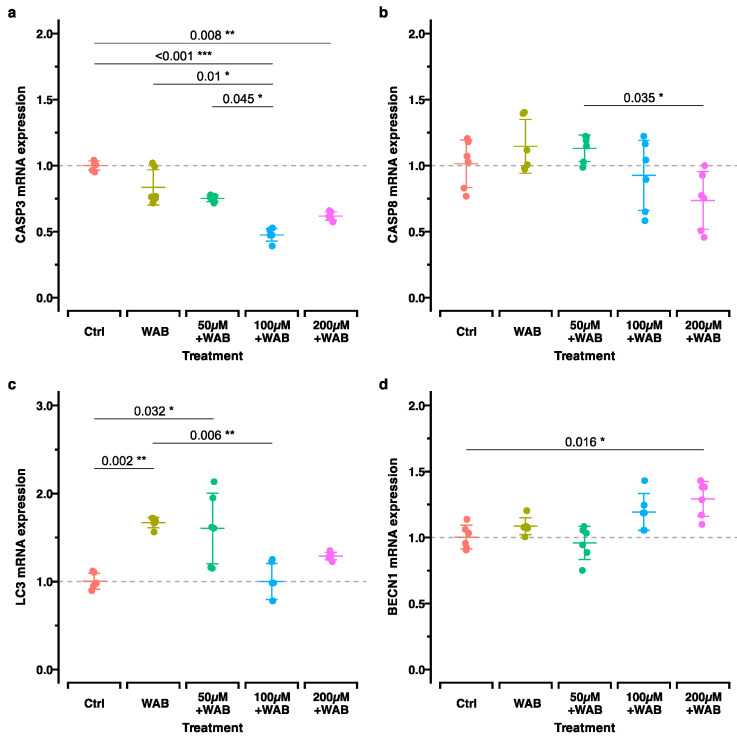
RT-qPCR results for autophagy (**a**,**b**)- and apoptosis (**c**,**d**)-related genes. Adjusted *p*-values based on multiple comparisons between each experimental treatment are shown. The groups are the same as in Figure 4. Adjusted *p*-values (*p*_adj_.) based on multiple comparisons between each experimental treatment are shown as follows: *, *p*_adj_. < 0.05; **, *p*_adj_. < 0.01; ***, *p*_adj_. < 0.001.

The apoptosis-related genes *CASP3* and *CASP8* exhibited either no regulation or a minimal tendency to be downregulated, which was more pronounced in the groups with previous H_2_O_2_ stimulation (Figure 5a,b). *CASP3* was significantly downregulated in response to compression following prestimulation with 100 μM or 200 μM H_2_O_2_ (Figure 5b). In contrast, the genes related to autophagy showed a slight, although a mostly statistically non-significant, upregulation tendency (Figure 5c,d). The *BECN1* expression was significantly increased after the application of the WAB method in the group previously stimulated with 200 μM H_2_O_2_. *MAP1LC3A*/*LC3* had a pronounced increase in expression following the application of the WAB method after pretreatment with 50 μM H_2_O_2_ (Figure 5c).

### 2.4. Assessment of Cell Viability and Proliferation Under Static Compression With and Without Oxidative Stress

**Figure 6:**
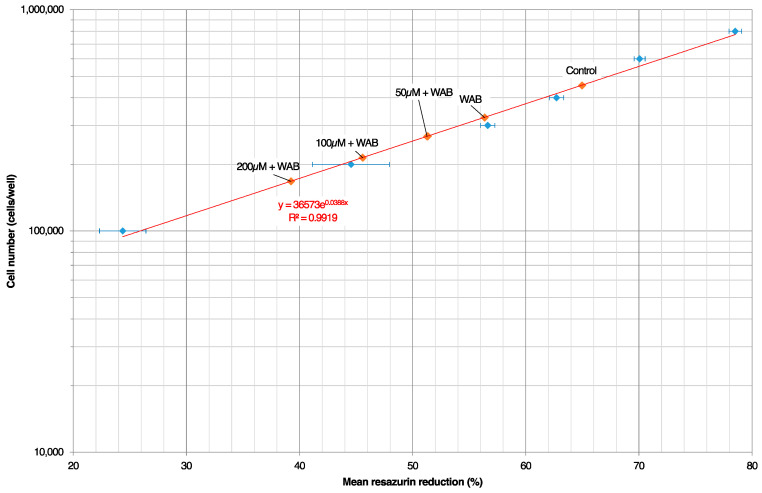
Resazurin-reduction-based growth curve. A standard curve was generated as described in the Section 4 to examine the cell growth during the experiments. Cells from the 5th passage were seeded in triplicate (100,000; 200,000; 300,000; 400,000; 600,000; and 800,000 cells/well). Exponential regression was used to calculate the standard curve (red line) (Microsoft Excel). Cellular growth of the hPDLCs in the different experimental conditions is shown with red diamonds (♦) on the fitted curve, and data from the standard curve are shown with blue diamonds (♦).

**Figure 7:**
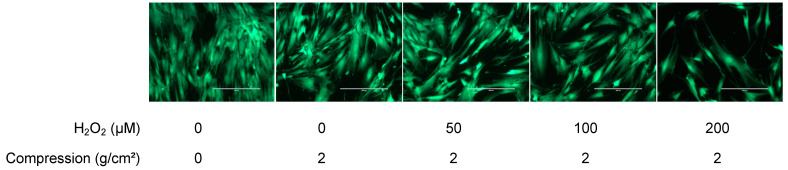
Results of the live/dead cell staining from the WAB in vitro model, with/without H_2_O_2_ stimulation for a qualitative assessment of the cell viability of the cells in different experimental groups. Green cells indicate viability, and unattached dead cells are either washed away or stained red (Scale bar: 200 μm).

Next, we analyzed the cell proliferation in the hPDLCs treated with H_2_O_2_ and subjected to static compression via calculations of the resazurin reduction (Figure 6). These experiments indicated a reduced proliferation in the groups pre-exposed to H_2_O_2_, with a more pronounced effect in the groups that received both OTM and H_2_O_2_ stimulation. Similar results were obtained through live/dead staining of the cells (Figure 7).

## 3. Discussion

The periodontal ligament (PDL) is a specialized connective tissue that plays a crucial role in anchoring the teeth within the alveolar bone, transmitting occlusal forces, providing sensory feedback, and contributing to the repair and regeneration of the periodontal tissues [31]. Its functionality is closely linked to its multi-potential progenitor cells, as well as its diverse cellular composition. Among its different cell types, PDL fibroblasts play a significant functional role, especially in the process of responding to mechanical forces [29,32,33,34]. These fibroblasts are crucial not only for maintaining the stability and health of the periodontium but also in mechanosensing processes, including those induced by orthodontic forces [29]. Depending on the type of force applied, they either support anabolic or catabolic processes. The compression side during orthodontic tooth movement (OTM) is characterized by a pronounced inflammatory response, increased bone resorption activity, reduced proliferation, and the activation of cell-destiny-related pathways. Excessive ROS accumulation, such as that seen in periodontal disease, is also associated with catabolic events [35,36]. Herein, we explored the influence of oxidative stress (OS) exposure on hPDLCs immediately after ROS stimulation using H_2_O_2_, as well as an additional 24 h post-incubation.

Additionally, we investigated how pre-existing OS affects the cellular physiology related to OTM, particularly in response to a compressive force. In this study, we focused on gene/substance expression related to inflammation (*IL6*, *CXCL8*/*IL8*, *PTGS2*/*COX2*, PGE2), bone remodeling (*RUNX2*, *BGLAP*, *TNFRSF11B*/*OPG*), autophagy (*MAP1LC3A*/*LC3*, *BECN1*), and apoptosis (*CASP3*, *CASP8*) and analyzed these at the transcriptional, translational, and activity levels via RT-qPCR and/or ELISA. While our results generally confirm the catabolic influence of OS, they also demonstrate the long-lasting altering effects of ROS exposure on gene expression. Additionally, our findings indicate that ROS exposure affects the mechanosensing processes during OTM, emphasizing the need for caution when treating older patients with pre-existing chronic inflammatory conditions.

### 3.1. Inflammation

Herein, we investigated the expression of the inflammatory markers *IL6*, *CXCL8*/*IL8*, *PTGS2*/*COX2*, and PGE2 in hPDLCs in response to OS and a static compressive force, both known to trigger inflammatory responses. These markers were selected due to their pivotal roles in the inflammatory cascade and their relevance to both periodontal pathology and OTM [17,37,38,39,40,41,42]. In this regard, studies have shown an increase in, for example, IL6 in gingival crevicular fluid during orthodontic tooth movement [43,44]. As such, we decided to track the regulation of IL6 at both the gene and protein expression levels.

#### 3.1.1. H_2_O_2_ Stimulation—Direct vs. 24 h Post-Incubation

It is known that increased OS and exposure to H_2_O_2_ can lead to the elevated expression of inflammatory markers [45]. In line with this, the herein-investigated markers *IL6* and *PTGS2*/*COX2* showed dose-dependent upregulation directly after H_2_O_2_ stimulation. On the other hand, contrary to the findings from literature [46], *CXCL8*/*IL8* decreased directly after H_2_O_2_ stimulation. In this study, we also showed that changes in gene expression were still visible 24 h after H_2_O_2_ stimulation, although to a lesser extent. The results presented herein indicate that H_2_O_2_ exposure alters the pro-inflammatory response; however, they also demonstrate its long-lasting effect. Further studies on the relationship of OS and pro-inflammatory markers at the cellular and molecular levels are needed to strengthen this evidence base.

#### 3.1.2. H_2_O_2_ Stimulation Followed by Static Compression

Although not statistically significant, slight gene expression upregulation in response to a static compressive force could be observed for *PTGS2*/*COX2* and *CXCL8*/*IL8.* This is in line with published studies [47,48,49,50,51,52,53,54,55]. Nevertheless, preincubation with H_2_O_2_ induced more pronounced dose-dependent upregulation of *PTGS2*/*COX2* and *CXCL8*/*IL8* gene expression, demonstrating an enhancing pro-inflammatory effect of ROS stimulation. The PGE2 concentration in the corresponding supernatants followed the *PTGS2*/*COX2* expression pattern as determined using RT-qPCR, which is consistent with its known role as a key enzyme involved in PGE2 synthesis [51,52,53,54]. Contrary to the findings in the literature [48,49,50,56], *IL6* gene expression showed slight downregulation, which was generally confirmed using IL6 ELISA, with the exception of prestimulation with 200 μM H_2_O_2_, which might indicate an altered inflammatory response to a higher exposure to OS. These results confirm an alteration in the inflammatory response, highlighting the complex interplay between OS and mechanical stimuli in regulating key inflammatory mediators.

### 3.2. Bone Remodeling

hPDLCs play an important role in activating molecular pathways related to both bone formation and resorption [57]. Herein, we analyzed the expression of the osteogenic marker genes *RUNX2*, *BGLAP*, and *TNFRSF11B*/*OPG* in the hPDLCs. *RUNX2* [58,59] is one of the essential transcription factors during bone development, also known to directly regulate the expression of *BGLAP* and *TNFRSF11B*/*OPG*, highlighting their interconnected roles in bone physiology [60]. *BGLAP* is a non-collagenous protein secreted by osteoblasts and is involved in bone mineralization and calcium ion homeostasis [61]. *TNFRSF1B*/*OPG* acts as a decoy receptor for RANKL (receptor activator of nuclear factor kappa B ligand), inhibiting its interaction with RANK (receptor activator of NF-kappaB) and thereby preventing osteoclast differentiation and bone resorption [62].

#### 3.2.1. H_2_O_2_ Stimulation—Direct vs. 24 h Post-Incubation

Our results showed a moderate upregulation of *RUNX2* directly after stimulation with the lower dose of H_2_O_2_ (50 μM) or no change with the higher doses (100/200 μM). Recently published studies [20,24] reported the downregulation of *RUNX2* in hPDLCs directly after stimulation with 100 μM H_2_O_2_ for 24 h. However, in both studies, the cells were precultured in an osteogenic induction medium before H_2_O_2_ stimulation, which might explain the difference. Nevertheless, according to our results, downregulation of *RUNX2* was measured 24 h post-incubation, showing delayed negative influence on osteogenesis. Similarly, 24 h post-incubation downregulation was observed in *BGLAP* gene expression in groups treated with 100/200 μM H_2_O_2_. In contrast to these findings, *TNFRSF11B*/*OPG* showed immediate dose dependent downregulation with recovery effect 24 h post-incubation.

#### 3.2.2. H_2_O_2_ Stimulation Followed by Static Compression

There are limited research data examining the regulation of *RUNX2* after the stimulation of hPDLCs with a static compressive force using the WAB in vitro model [63,64]. All of the studies identified so far reported either the downregulation of *RUNX2* gene expression or no stimulation at all within the first 24 h of the application of a static compressive force. In contrast, herein, *RUNX2* was upregulated by the application of a compressive force, while in cases of previous H_2_O_2_ exposure, no change in gene expression was observable. Similarly, when comparing the reaction to static compression between the cells with and without previous H_2_O_2_ exposure, *BGLAP* was also significantly less expressed in the cells treated with 100 μM and 200 μM H_2_O_2_ compared to the cells that just received the force. Our results showed a significant decrease in *TNFRSF11B*/*OPG* in the cells pretreated with 100 μM and 200 μM of H_2_O_2_ and then subjected to the compression force. In contrast, no significant change in *TNFRSF11B*/*OPG* was observed in the cells that underwent the mechanical force, which is in accordance with previous results [65]. Nevertheless, different studies have reported different expressions of *TNFRSF11B*/*OPG* in hPDLCs subjected to mechanical compression, examining different magnitudes and durations of compression [54,66,67,68,69].

### 3.3. Autophagy and Apoptosis

OS has been shown to be involved in various biological and cellular pathways, including apoptosis and autophagy [15]. The overproduction of ROS can have destructive effects on the structure of cell organelles and biomolecules [15], triggering cell-destiny-related pathways. Recent studies have found that autophagy protects PDLCs from apoptosis and promotes recovery in an inflammatory microenvironment. In periodontitis, autophagy helps to eliminate the periodontal pathogen infection and modulates the immune inflammatory response [70]. Similarly, these pathways are also activated by mechanosensing [71,72]. To obtain a better insight into these relationships, we focused on *CASP3* and *CASP8*, with both playing an important role in cell apoptosis, and the autophagy-related markers *BECN1* and *MAP1LC3A*/*LC3* [73]. While *BECN1* plays a crucial role in the early stages of autophagy, *MAP1LC3A*/*LC3* is an essential marker in the final stages, specifically during autophagosome formation [74].

#### 3.3.1. H_2_O_2_ Stimulation—Direct vs. 24 h Post-Incubation

Previous studies have shown increased rates of apoptosis and autophagy in PDLCs and periodontal ligament stem cells following OS stimulation [11,26,27,75]. Although the upregulation of the autophagy-related genes *MAP1LC3A*/*LC3* and *BECN1* was not always statistically significant herein, a general tendency toward upregulation was observed after H_2_O_2_ stimulation, aligning with previous findings [26]. However, these effects diminished in most cases 24 h post-incubation. In contrast, no clear expression pattern was observed for the apoptosis-related genes *CASP3* and *CASP8*. Given the limited research on hPDLCs regarding OS, autophagy, and apoptosis, further studies are essential to clarify the precise impact of OS on these cellular mechanisms [73].

#### 3.3.2. H_2_O_2_ Stimulation Followed by Static Compression

Studies have reported contradictory effects of a compressive force on autophagy-related markers. One study reported a decrease in LC3II/I and BECN1 protein after 24 h of a 1.5 g/cm^2^ compressive force [67], whereas another reported an increase in BECN1 protein and the ratio of LC3II/I after 24 h of stimulation with a 2 g/cm^2^ compressive force in hPDLCs [76], along with elevated CASP3 protein levels. Herein, the apoptosis-related genes exhibited either no regulation or a minimal downregulation tendency, which was slightly more pronounced in groups with previous H_2_O_2_ stimulation. On the contrary, the genes related to autophagy seemed to show a slight, although statistically non-significant in most cases, upregulation tendency.

### 3.4. This Study’s Strengths/Limitations and Future Perspectives

OS has not been thoroughly investigated in relationship to mechanosensing in hPDLCs. In this study, we combined two established models: one using H_2_O_2_ to simulate OS and the WAB model as the other to mimic orthodontic static compressive forces. To our knowledge, this represents the first attempt to investigate the combined effects of static compression forces and OS on hPDLCs.

Direct exposure of cells to oxidative chemicals like H_2_O_2_ is a commonly used method for simulating OS [77]. H_2_O_2_, an ROS frequently generated during inflammation, including periodontal disease [7,78,79,80], makes this approach particularly relevant to studying OS in PDLCs. This method is supported by well-established protocols, allowing for precise control over the concentration and exposure time [81]. For a more physiological simulation of OS, alternative models involving the knockout or suppression of antioxidant defense genes have been proposed [82]. While such approaches can provide a more realistic representation of chronic OS, they come with certain limitations. Many genes have multiple functions, meaning that knocking out a single gene can trigger unexpected phenotypic changes unrelated to OS. Additionally, cells may activate compensatory pathways to offset the loss of gene function, potentially obscuring the true effects of the knockout. Furthermore, permanent gene knockout complicates the study of time-dependent processes, which was particularly relevant in our study, as we aimed to simulate the “recovery effect” following periodontal therapy.

While in vitro models have limitations in replicating the biological complexity of living organisms [83], this study lays a foundation for more sophisticated analyses by focusing on key mechanistic insights in a controlled setting. We specifically used hPDLCs in a 2D cellular monolayer, providing a direct view of their response to OS and compressive forces. However, in living tissues, interactions with neighboring cells, the extracellular matrix, and signaling molecules [84,85] influence these processes. Therefore, future studies, such as those using coculture or three-dimensional (3D) models, would further enhance our understanding by mimicking the in vivo environment more closely, allowing for intercellular communication and a more physiologically relevant structure [86]. Our study relied on a qPCR-based analysis of *MAP1LC3A*/*LC3* and *BECN1* as markers to assess cell autophagy. While these markers are widely known autophagy regulators [87,88], their roles in other cellular pathways and the lack of direct measurement of autophagic flux limit the specificity of our conclusions. Future studies including such direct assays will provide a more comprehensive evaluation of autophagic activity [89]. Incorporating additional functional assays, such as TUNEL assays for apoptosis detection [90], would provide a more comprehensive understanding of how oxidative stress and mechanical force impact hPDLC physiology. Additionally, a broader exploration at the gene and protein levels, incorporating cells from multiple donors of varying ages and sexes, as well as further in vivo studies, could provide valuable insights into the variability in these responses. Such studies would help account for individual differences in biology and physiology, bringing us closer to understanding how these factors contribute to OTM and related bone remodeling processes in vivo. Future research should include animal models to validate our findings in a more physiologically relevant context, enabling a deeper understanding of the interplay between OS, gene regulation, and tissue remodeling during orthodontic treatment.

## 4. Materials and Methods

### 4.1. Primary Cell Culture

Human PDLCs were isolated from the middle third of the roots of caries-free molars from a healthy 18-year-old male that were extracted due to orthodontic reasons. Written informed consent from the patient or his legal custodian was obtained. This study was conducted in accordance with the Declaration of Helsinki, and the study protocol was approved by the ethics committee of the Ludwig-Maximilians-Universität München (project number 21-0931).

HPDLCs were obtained from tissue samples utilizing a modified explant technique [91] and were cultured in Dulbecco’s Modified Eagle’s Medium/Nutrient Mixture F-12 Ham (DMEM/F-12) (D6421; Sigma-Aldrich, St. Louis, MO, USA) supplemented with 10% FBS (F7524; Sigma-Aldrich, St. Louis, MO, USA), 2% MEM vitamins (M6895; Biochrom, Berlin, Germany), and 1% antibiotic/antimycotic (15240-062; Life Technologies, Carlsbad, CA, USA). The cells were maintained in a humidified environment with 5% CO_2_ at 37 °C and passaged at regular intervals using 0.05% trypsin–EDTA solution (L2143; Biochrom, Berlin, Germany). Cells from passages 5 and 6 were used in all of the experimental procedures.

### 4.2. H_2_O_2_ Concentration Gradient Selection

To identify the appropriate concentration of H_2_O_2_ for the experiment, cell viability, cytotoxicity, and apoptosis were analyzed after the stimulation of the hPDLCs with different H_2_O_2_ concentrations (9681.4; Carl Roth GmbH + Co. KG, Karlsruhe, Germany). The cells were seeded at a density of 1.5 × 10^5^ cells/well into 6-well plates (657160; Greiner Bio One, Frickenhausen, Germany) in triplicate and incubated overnight in a CO_2_ incubator (5% CO_2_, 37 °C, humidified atmosphere). On the next day, the cells were stimulated with H_2_O_2_ concentrations of 20 µM, 50 µM, 100 µM, 200 µM, and 500 µM [11,20,24] and incubated for 24 h. Wells containing the cell culture medium without H_2_O_2_ were considered as medium controls. The cytotoxicity of H_2_O_2_ in terms of cell viability was monitored using a resazurin-based assay, as previously published [92]. Cell viability and apoptosis were visually assessed as described below.

*Quantitative assessment of cell viability*/*cytotoxicity*: Cell viability and cytotoxicity were assayed using a resazurin assay as previously described [92] with one minor modification: the cells were incubated with resazurin-containing medium for 2 h instead of 3 h. The cell viability was then calculated as the normalized resazurin reduction relative to the control group.

*Qualitative assessment of cell viability and apoptosis using fluorescence microscopy*: Following the resazurin test, all of the wells were washed twice with phosphate-buffered saline (PBS, RNBL5636; Sigma-Aldrich, St. Louis, MO, USA). From each experimental group, two wells were used for live/dead cell staining, and one well was used for apoptosis detection. For apoptosis detection, the CellEvent™ Caspase-3/7 Green Reagent kit (R37111; Life Technologies, Carlsbad, CA, USA) was employed according to the manufacturer’s instructions. The cell viability of the hPDLCs at all concentrations was evaluated using a live/dead cell staining kit (L3224; Invitrogen/ThermoFisher Scientific, Carlsbad, CA, USA) according to the manufacturer’s instructions. After 30 min of incubation at room temperature in the dark, fluorescence microscopy was conducted (EVOS^®^*fl*, Invitrogen, Carlsbad, CA, USA) for both the live/dead and caspase 3/7 apoptosis tests. Photographs of randomly selected fields were captured using 10× and 20× microscope objectives.

### 4.3. Effect of Oxidative Stress Induction on Gene Expression in “Direct” and “Recovery” Setups

To assess the effect of H_2_O_2_ on the cells, we defined two setups: one for investigating the effect of H_2_O_2_ treatment directly after 24 h (the “direct” setup) and another one after an additional 24 h post-incubation (the “recovery” setup) (Figure 3a). For this purpose, the cells were seeded and incubated overnight as described above. The next day, the cell culture medium was replaced with culture medium containing the determined H_2_O_2_ concentrations (50 µM, 100 µM, or 200 µM) in triplicate (*n* = 3). Wells which received the cell culture medium without H_2_O_2_ were considered controls. Cell lysates of the “direct” setup were collected after 24 h of incubation from each well using 750 µL RNA lysis buffer (R0160-1-50; Zymo, Irvine, CA, USA) according to the manufacturer’s instructions and stored at −80 °C for further RT-qPCR tests (Section 4.5). For the “recovery” setup, fresh normal cell culture medium was added to all of the wells. Twenty-four hours post-incubation, the cell lysates were prepared and stored according to the same method.

### 4.4. Application of Compressive Force During the H_2_O_2_ “Recovery” Phase

To investigate the effect of H_2_O_2_ recovery and/or the compression force, the cells were evenly seeded as described above in two identical setups of experiments, each consisting of five experimental conditions: One setup was then used for cell viability and proliferation testing, and the other one was used for RT-qPCR (Section 4.5) and ELISA (Section 4.6). For each experimental group, three biological replicates were allocated (*n* = 3). All of the plates were incubated overnight as described above. The cells were stimulated with or without 50 µM, 100 µM, and 200 µM H_2_O_2_ the following day and incubated for 24 h.

#### 4.4.1. Application of Force with the WAB Model

After 24 h of stimulation with or without the abovementioned concentrations of H_2_O_2_ in a CO_2_ incubator, a physiological compressive force of 2.0 g/cm^2^ was applied to the cells for a further 24 h (Figure 4a). Immediately prior to the application of force, the old culture medium was removed, and all of the wells were washed twice with PBS. Fresh culture medium was then added (2.5 mL/well). The compressive force was applied using a sterile glass disc and a small container with lead granules, as described in previously [92].

#### 4.4.2. Cell Proliferation and Cell Viability

As previously described by Janjic Rankovic et al. [92], a resazurin standard curve was prepared as follows. Cells from the 5th passage were seeded in triplicate (100,000; 200,000; 300,000; 400,000; 600,000; and 800,000 cells per well). “*Percentage reduction of resazurin*” was calculated according to the manufacturer’s instructions, and a standard curve (cell number vs. percentage reduction in resazurin) was established using exponential regression (Microsoft Excel for Windows 365 MSO Version 2404, Microsoft Corporation, Redmond, WA, USA) (Figure 6). After the application of force with the WAB method (Section 4.4.1) the compression setup was carefully disassembled, and the resazurin assessment was conducted as described above. The cell number per well was calculated using the prepared standard curve.

Additionally, cell viability was assessed qualitatively using the abovementioned live/dead cell staining kit according to the manufacturer’s instructions. After the release of the force and the removal of the resazurin test supernatants, all of the wells were washed twice with PBS, and microphotographs were captured as previously outlined (Section 4.2)

#### 4.4.3. Sample Preparation for RT-qPCR and ELISA

After 48 h of cellular stimulation, the cell culture supernatants from the respective setup were collected. Afterwards, the adherent cells were washed twice with sterile PBS. Cell lysates were then prepared as explained above (Section 4.3). In between, the collected cell culture supernatants were centrifuged and stored at −80 °C for the ELISA analysis [93].

### 4.5. Reverse Transcription Quantitative Polymerase Chain Reaction (RT-qPCR)

An analysis of *PTGS2*/*COX2*, *IL6*, *CXCL8*/*IL8*, *RUNX2*, *TNFRSF11B*/*OPG*, *BGLAP*, *CASP3*, *CASP8*, *MAP1LC3A*/*LC3*, and *BECN1* gene expression following H_2_O_2_ stimulation and/or the application of compressive force was carried out for all experimental groups according to previously described protocols [92,93]. Below is an overview of the sample preparation and quantitative RT-PCR (RT-qPCR) process. Additionally, a checklist according to the “*Minimum Information for Publication of Quantitative Real-Time PCR Experiment*” (MIQE) guidelines [94] is available in Supplementary Table S2.1.

*Total RNA preparation and cDNA synthesis*: RNA isolation and cDNA synthesis were conducted utilizing the QuickRNA™ MicroPrep Kit (R1051; Zymo, Irvine, CA, USA) and the SuperScript™ IV First-Strand Synthesis System (18091050, Thermo Fisher Scientific, Waltham, MA, USA), respectively, and based on previously published data [92,93].

*PCR primer selection*: As previously published [92], the primer sequences for all of the genes were sourced from public databases and were tested in silico according to the MIQE guidelines [95] (Supplementary Table S2.1). Unmodified primers were acquired from a commercial source (TIB Molbiol Syntheselabor GmbH, Berlin, Germany). Gradient PCR using the qPCR cycling program as specified in the MIQE checklist was performed to determine the optimal annealing temperatures. The primer specificity was confirmed through agarose gel electrophoresis. To assess the efficiencies of each primer, serial dilutions of cDNA were used to create specific standard curves. These data were then quantified using the LightCycler^®^ 480 with the primer pairs as described in Supplementary File S2.

**Figure 8:**
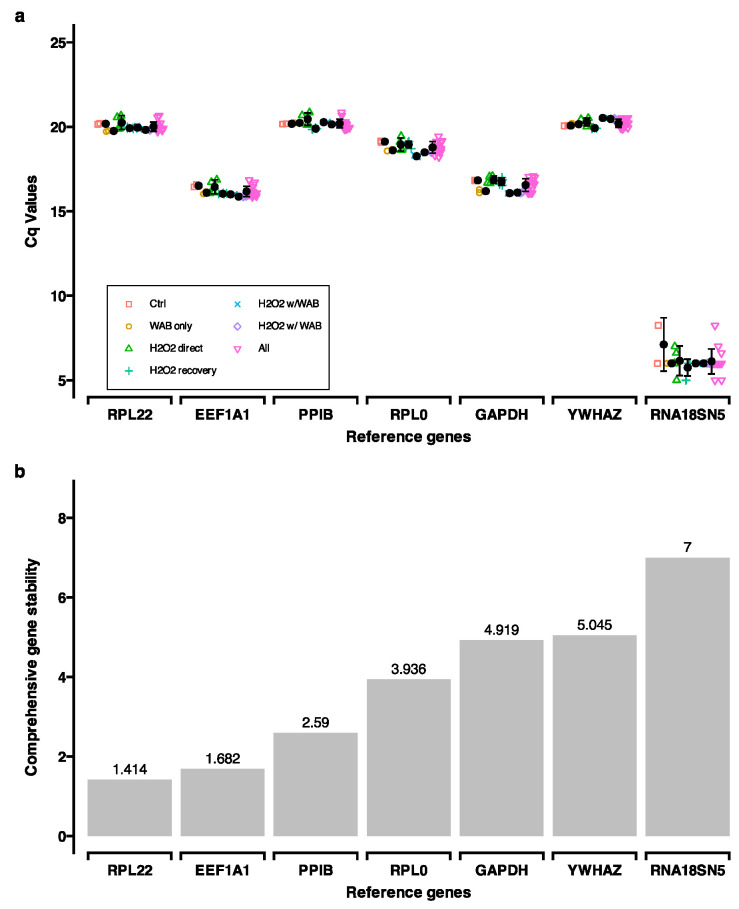
Reference gene selection was undertaken using RefFinder. (**a**) Cq values for the panel of reference genes. Six quantitative polymerase chain reaction (qPCR) runs were analyzed, representing three biological replicates and two technical replicates each (Supplementary Table S2.3). (**b**) Analysis of comprehensive gene stability for the panel of reference genes. Lower values indicate higher gene stability (Supplementary File S2).

*Reference gene selection*: A panel of reference genes (*EEF1A1*, *GAPDH*, *PPIB*, *RNA18SN5*, *RPL0*, *RPL22*, and *YWHAZ*) was selected from public sources [96,97]. cDNA from the experimental groups “Control”, “100 µM H_2_O_2_”, “100 µM H_2_O_2_ + WAB”, “50 µM H_2_O_2_”, “50 µM H_2_O_2_ + WAB”, “WAB”, “50 µM H_2_O_2_” (directly after 24 h of stimulation), and “100 µM H_2_O_2_” (directly after 24 h of stimulation) was used to evaluate the reference genes. Gene-specific primers were then used to conduct RT-qPCR (Supplementary File S2). RefFinder [98,99] was used to analyze the raw Cq values (Supplementary Table S2.2). This web-based tool integrates four different algorithms (BestKeeper [100], NormFinder [101], geNorm [102], and the comparative ΔCt method [103]) to compare and rank candidate reference genes. Based on the rankings, the most stable genes (*RPL22* and *EEF1A1*) were used as the reference genes in RT-qPCR (Figure 8).

*Quantitative PCR*: The LightCycler^®^ 480 SYBR Green I Master Kit (04887352001; Roche Diagnostics GmbH, Mannheim, Germany) was used to conduct RT-qPCR according to the manufacturer’s protocol (5 µL of cDNA (1:10 prediluted) in each PCR reaction). The details are mentioned in the MIQE checklist (Supplementary File S1). The PCR primer specifications are summarized in Table 1.

*Gene expression calculation*: After averaging (geometric mean) the reference genes (*RPL22* and *EEF1A1*) for a given WAB/H_2_O_2_ concentration combination, the 2^−ΔΔCq^ method was applied for quantification of the gene expression [102,104]. For each experimental group, six qPCR reactions were analyzed: two technical replicates from each of the three biological replicates (N = 3, *n* = 6).

**Table 1:** Specification of the PCR primers used for gene quantification.

Gene	GenBank Accession Number	Primer Sequence (f: 5′-Forward Primer-3′; r: 5′-Reverse Primer-3′)	Annealing Temp. (°C)	Amplicon Size (bp)	Reference
*PTGS2*/*COX2*	NM_000963.4	f: AAGCCTTCTCTAACCTCTCC r: GCCCTCGCTTATGATCTGTC	58	234	[92,105]
*IL6*	NM_000600.5	f: TGGCAGAAAACAACCTGAACC r: TGGCTTGTTCCTCACTACTCTC	58	168	[92,105]
*CXCL8*/*IL8*	NM_000584.4	f: CAGAGACAGCAGAGCACACAA r: TTAGCACTCCTTGGCAAAAC	55	170	[106]
*RUNX2*	NM_001015051.4	f: GCGCATTCCTCATCCCAGTA r: GGCTCAGGTAGGAGGGGTAA	58	176	[92,107]
*BGLAP*	NM_199173.6	f: AGCGAGGTAGTGAAGAGAC r: GAAAGCCGATGTGGTCAG	64	142	[108]
*BECN1*	NM_003766.5	f: AGGTTGAGAAAGGCGAGACA r: AATTGTGAGGACACCCAAGC	58	196	[109]
*MAP1LC3A*/*LC3*	NM_032514.4	f: CGTCCTGGACAAGACCAAGT r: TCCTCGTCTTTCTCCTGCTC	58	183	[109]
*CASP3*	NM_004346.4	f: TGGAGGCCGACTTCTTGTAT r: ACTGTTTCAGCATGGCACAA	58	111	[110]
*CASP8*	NM_001228.5	f: GGAGGAGTTGTGTGGGGTAA r: CCTGCATCCAAGTGTGTTCC	58	207	[111]
*TNFRSF11B*	NM_002546.4	f: TCAAGCAGGAGTGCAATCG r: AGAATGCCTCCTCACACAGG	64	342	[112]
*EEF1A1*	NM_001402.6	f: CCTGCCTCTCCAGGATGTCTAC r: GGAGCAAAGGTGACCACCATAC	61	105	[93,97]
*PPIB*	NM_000942.5	f: TTCCATCGTGTAATCAAGGACTTC r: GCTCACCGTAGATGCTCTTTC	55	88	[93,97]

### 4.6. Enzyme-Linked Immunosorbent Assay (ELISA)

Cell culture supernatants from all of the wells were collected for ELISA as described. The IL6 and PGE2 concentrations in the cell culture supernatants were determined through ELISA (IL6: DuoSet human IL6 ELISA kit, DY206-05, R&D Systems, Minneapolis, MN, USA; PGE2: PGE2 High-Sensitivity ELISA kit, ADI-931-001, Enzo Life Sciences AG, Lausen, Switzerland) on a Varioscan microplate reader (Thermo Electron Corporation, Vantaa, Finland). Each marker molecule and experimental condition combination was tested in three biological replicates, with each measured twice. The results were reported as “pg per 100,000 cells”, using the well-specific cell numbers determined previously.

### 4.7. Statistics

Descriptive statistics on the gene expression and ELISA results are reported as the mean and standard deviation (SD), median, and minimum/maximum. All of the calculations were based on three biological replicates, with two technical replicates for each gene/experimental condition combination. For each gene locus and marker molecule, differences between the different magnitudes of tensile strain and durations were evaluated using the Kruskal–Wallis test followed by multiple comparisons with Bonferroni correction applied (*p*_adj._). All of the statistical procedures were carried out using IBM SPSS Statistics 29 (IBM Corp., Armonk, NY, USA) and were two-tailed, considering *p*_adj._ values < 0.05 as significant.

## 5. Conclusions

The results of this in vitro study not only confirm the catabolic effects of OS but also highlight the prolonged influence of ROS exposure (24 h post-incubation) on the expression of genes that regulate inflammation, bone metabolism, and cell fate in hPDLCs. Furthermore, our findings suggest that ROS alters mechanosensing in hPDLCs during the application of static compression force, directly impacting the expression of these key genes.

This alteration could potentially shift the balance between bone resorption and formation, leading to complications in orthodontic treatment. Understanding these mechanisms is essential for developing personalized treatment options that align with the unique biological characteristics of each patient. Additionally, our study highlights the need to create strategies aiming to reduce OS, thereby enhancing the effectiveness and safety of orthodontic treatments. By addressing these factors, we can improve and ensure more tailored successful orthodontic care, particularly in elderly patients or patients with chronic inflammatory diseases, where ROS levels are known to be elevated.

## Data Availability

All of the authors confirm that all related data supporting the findings of this study are given in the article and its Supplementary Materials.

## Supplementary Materials

The following supporting information can be downloaded at https://www.mdpi.com/article/10.3390/ijms252413513/s1.

## Abbreviations

*BECN1*	Beclin 1
*BGLAP*	Bone gamma-carboxyglutamate protein
*CASP3*	Caspase 3
*CASP8*	Caspase 8
*CXCL8*/*IL8*	C-X-C Motif Chemokine Ligand 8 (aka IL8 (interleukin-8))
ELISA	Enzyme-linked immunosorbent assay
FC	Fold change
H_2_O_2_	Hydrogen peroxide
hPDLCs	Human periodontal ligament cells
IL6	Interleukin 6
*MAP1LC3A*/*LC3*	Microtubule-Associated Protein 1 Light Chain 3 Alpha (aka LC3)
MIQE	Minimum Information for Publication of Quantitative Real-Time PCR Experiment
OS	Oxidative stress
OTM	Orthodontic tooth movement
*P*_adj._	Adjusted *p*-value
PGE2	Prostaglandin E2
PTGS2/COX2	Prostaglandin Endoperoxide Synthase 2 (aka COX2 (cyclooxygenase 2))
ROS	Reactive oxygen species
RT-qPCR	Reverse transcription quantitative polymerase chain reaction
*RUNX2*	RUNX Family Transcription Factor 2
*TNFRSF11B*/*OPG*	TNF Receptor Superfamily Member 11b (aka OPG (osteoprotegerin))
