# Supplementary material for: Investigation of Impact of Oxidative Stress on Human Periodontal Ligament Cells Exposed to Static Compression
Source: Int J Mol Sci. 2024 Dec 17;25(24):13513. doi: 10.3390/ijms252413513 (PMC11678643; doi:10.3390/ijms252413513)

Supplement 2 to manuscript

"Investigation of Oxidative Stress Impact on Human Periodontal Ligament Cells Exposed to Static Compression"

URL: https://www.ciidirsinaloa.com.mx/RefFinder-master/ (2024-04-26)

RefFinder is a user-friendly web-based comprehensive tool developed for evaluating and screening reference genes from extensive experimental datasets. It integrates the currently available major computational programs (**geNorm**, **Normfinder**, **BestKeeper**, and **the comparative ΔCt method**) to compare and rank the tested candidate reference genes. Based on the rankings from each program, it assigns an appropriate weight to an individual gene and calculated the geometric mean of their weights for the overall final ranking.

Citation: **F Xie, P Xiao, D Chen, L Xu, B Zhang. 2012. miRDeepFinder: a miRNA analysis tool for deep sequencing of plant small RNAs. Plant molecular biology 80 (1), 75-84.**

References

1. BestKeeper: Pfaffl MW, Tichopad A, Prgomet C, Neuvians TP. 2004. Determination of stable housekeeping genes, differentially regulated target genes and sample integrity: BestKeeper--Excel-based tool using pair-wise correlations. Biotechnology letters 26:509-515.
2. NormFinder: Andersen CL, Jensen JL, Orntoft TF. 2004. Normalization of real-time quantitative reverse transcription-PCR data: a model-based variance estimation approach to identify genes suited for normalization, applied to bladder and colon cancer data sets. Cancer research 64:5245-5250.
3. Genorm: Vandesompele J, De Preter K, Pattyn F, Poppe B, Van Roy N, De Paepe A, Speleman F. 2002. Accurate normalization of real-time quantitative RT-PCR data by geometric averaging of multiple internal control genes. Genome biology 3:RESEARCH0034.
4. The comparative delta-Ct method: Silver N, Best S, Jiang J, Thein SL. 2006. Selection of housekeeping genes for gene expression studies in human reticulocytes using real-time PCR. BMC molecular biology 7:33.

Contents

RAW DATA (CQ VALUES) 2
REFFINDER – SUMMARY 3
DELTA CT 4
BESTKEEPER 5
NORMFINDER 6
GENORM 7

Supplement 2: RefFinder results

Raw data (Cq values)

Treatment	PPIB	GAPDH	RPL22	YWHAZ	EEF1A1	RPL0	RNA18SN5
Control	20.16	16.81	20.21	20.05	16.44	19.17	8.24
Control	20.19	16.85	20.14	20.09	16.59	19.07	6.00
H_2O_2 100µM	19.95	16.53	19.88	19.94	16.02	19.13	5.00
H_2O_2 100µM	19.84	17.00	19.90	19.97	16.06	19.08	6.00
H_2O_2 100µM + WAB	20.13	16.13	19.80	20.43	15.86	18.50	6.00
H_2O_2 100µM + WAB	20.15	16.08	19.83	20.48	15.89	18.47	6.00
H_2O_2 50µM	19.90	16.59	19.99	19.90	16.08	18.71	6.00
H_2O_2 50µM	19.88	16.86	19.92	19.88	16.01	18.87	6.00
H_2O_2 50µM +WAB	20.28	16.08	19.85	20.53	16.00	18.21	6.00
H_2O_2 50µM +WAB	20.27	16.07	20.05	20.51	15.99	18.31	6.00
WAB	20.24	16.28	19.78	20.10	16.16	18.57	6.00
WAB	20.22	16.10	19.72	20.20	16.03	18.65	6.00
H_2O_2 100µM (direct)	20.10	16.68	19.88	20.16	16.10	18.62	5.00
H_2O_2 100µM (direct)	20.18	16.65	19.89	20.01	16.06	18.64	6.00
H_2O_2 50µM (direct)	20.67	17.08	20.56	20.44	16.72	19.44	6.61
H_2O_2 50µM (direct)	20.84	17.05	20.66	20.51	16.86	19.09	7.00

	Ctrl (N= 2)	WAB only (N= 2)	H_2O_2 direct (N= 4)	H_2O_2 recovery (N= 4)	H_2O_2 w/WAB (N= 2)	H_2O_2 w/WAB (N= 2)	All (N= 16)
PPIB							
Mean (SD)	20 (0.021)	20 (0.014)	20 (0.36)	20 (0.046)	20 (0.0071)	20 (0.014)	20 (0.26)
Median [Min, Max]	20 [20, 20]	20 [20, 20]	20 [20, 21]	20 [20, 20]	20 [20, 20]	20 [20, 20]	20 [20, 21]
GAPDH							
Mean (SD)	17 (0.028)	16 (0.13)	17 (0.23)	17 (0.22)	16 (0.0071)	16 (0.035)	17 (0.38)
Median [Min, Max]	17 [17, 17]	16 [16, 16]	17 [17, 17]	17 [17, 17]	16 [16, 16]	16 [16, 16]	17 [16, 17]
RPL22							
Mean (SD)	20 (0.049)	20 (0.042)	20 (0.42)	20 (0.048)	20 (0.14)	20 (0.021)	20 (0.27)
Median [Min, Max]	20 [20, 20]	20 [20, 20]	20 [20, 21]	20 [20, 20]	20 [20, 20]	20 [20, 20]	20 [20, 21]
YWHAZ							
Mean (SD)	20 (0.028)	20 (0.071)	20 (0.24)	20 (0.040)	21 (0.014)	20 (0.035)	20 (0.24)
Median [Min, Max]	20 [20, 20]	20 [20, 20]	20 [20, 21]	20 [20, 20]	21 [21, 21]	20 [20, 20]	20 [20, 21]
EEF1A1							
Mean (SD)	17 (0.11)	16 (0.092)	16 (0.41)	16 (0.033)	16 (0.0071)	16 (0.021)	16 (0.30)
Median [Min, Max]	17 [16, 17]	16 [16, 16]	16 [16, 17]	16 [16, 16]	16 [16, 16]	16 [16, 16]	16 [16, 17]
RPL0							
Mean (SD)	19 (0.071)	19 (0.057)	19 (0.39)	19 (0.19)	18 (0.071)	18 (0.021)	19 (0.35)
Median [Min, Max]	19 [19, 19]	19 [19, 19]	19 [19, 19]	19 [19, 19]	18 [18, 18]	18 [18, 19]	19 [18, 19]
RNA18SN5							
Mean (SD)	7.1 (1.6)	6.0 (0)	6.2 (0.87)	5.8 (0.50)	6.0 (0)	6.0 (0)	6.1 (0.74)
Median [Min, Max]	7.1 [6.0, 8.2]	6.0 [6.0, 6.0]	6.3 [5.0, 7.0]	6.0 [5.0, 6.0]	6.0 [6.0, 6.0]	6.0 [6.0, 6.0]	6.0 [5.0, 8.2]

RefFinder – Summary

Ranking Order (Better--Good--Average)

Method	1	2	3	4	5	6	7
Delta CT	RPL22	EEF1A1	PPIB	RPL0	GAPDH	YWHAZ	RNA18SN5
BestKeeper	PPIB	RPL22	YWHAZ	EEF1A1	RPL0	GAPDH	RNA18SN5
Normfinder	EEF1A1	RPL22	PPIB	RPL0	GAPDH	YWHAZ	RNA18SN5
Genorm	RPL22 | EEF1A1		RPL0	GAPDH	PPIB	YWHAZ	RNA18SN5
**Recommended comprehensive ranking**	**RPL22**	**EEF1A1**	**PPIB**	**RPL0**	**GAPDH**	**YWHAZ**	**RNA18SN5**

Comprehensive Ranking:

Genes	Geomean of ranking values
RPL22	1.41
EEF1A1	1.68
PPIB	2.59
RPL0	3.94
GAPDH	4.95
YWHAZ	5.05
RNA18SN5	7.00

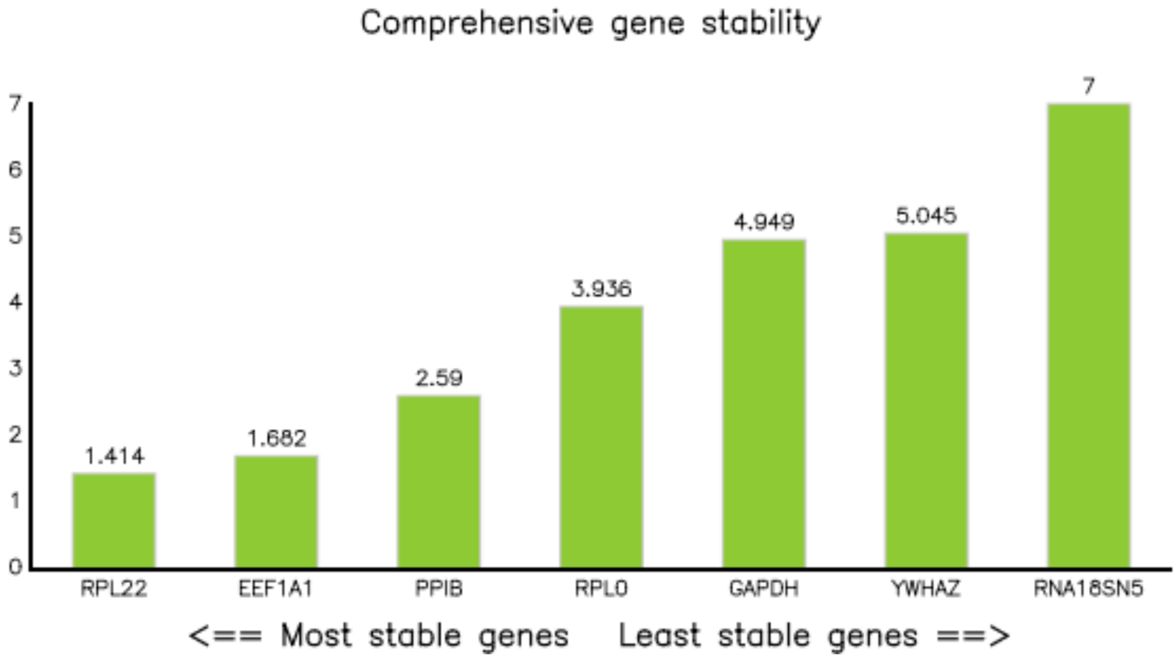

Supplement 2: RefFinder results

Delta CT

Genes	Average of STDEV
RPL22	0.30
EEF1A1	0.31
PPIB	0.35
RPL0	0.38
GAPDH	0.40
YWHAZ	0.43
RNA18SN5	0.68

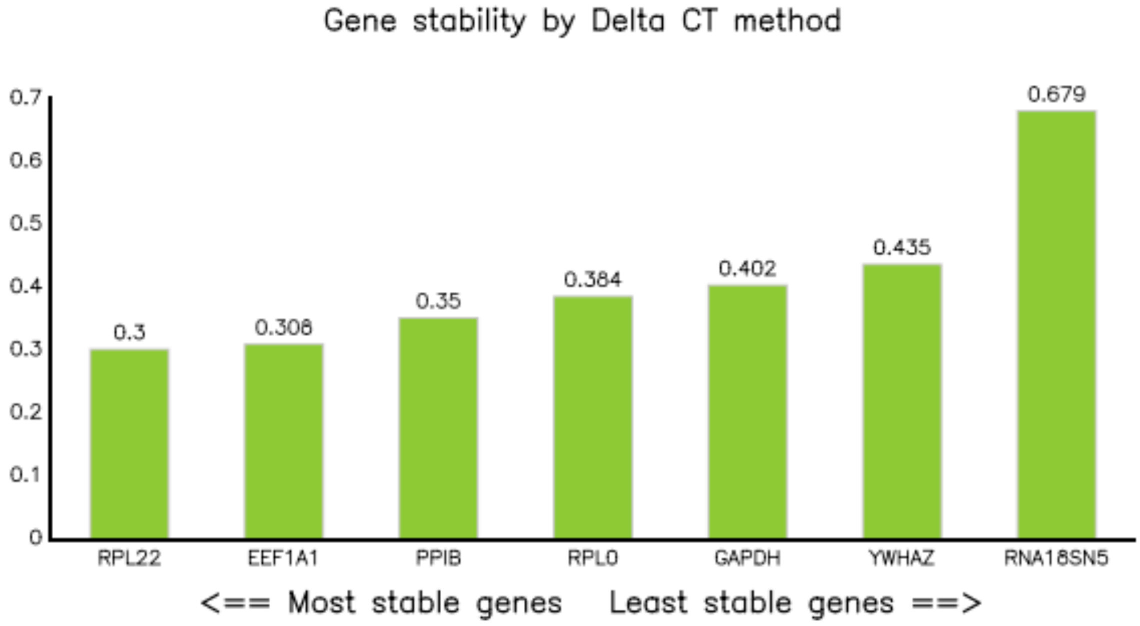

Supplement 2: RefFinder results

BestKeeper

CP data of housekeeping Genes by BEST KEEPER

	PPIB	GAPDH	RPL22	YWHAZ	EEF1A1	RPL0	RNA18SN5
n	16	16	16	16	16	16	16
geo Mean [CP]	20.19	16.55	20.00	20.20	16.18	18.78	6.08
AR Mean [CP]	20.19	16.55	20.00	20.20	16.18	18.78	6.12
min [CP]	19.84	16.07	19.72	19.88	15.86	18.21	5.00
max [CP]	20.84	17.08	20.66	20.53	16.86	19.44	8.24
std dev [+/- CP]	0.17	0.32	0.20	0.21	0.24	0.30	0.44
CV [% CP]	0.87	1.96	1.00	1.05	1.46	1.58	7.16
min [x-fold]	-1.27	-1.39	-1.22	-1.25	-1.25	-1.48	-2.11
max [x-fold]	1.57	1.45	1.58	1.26	1.61	1.58	4.48
std dev [+/- x-fold]	1.13	1.25	1.15	1.16	1.18	1.23	1.35

Pearson correlation coefficient (r) by BEST KEEPER

	PPIB	GAPDH	RPL22	YWHAZ	EEF1A1	RPL0	RNA18SN5
GAPDH	0.157	-	-	-	-	-	-
p-value	0.561	-	-	-	-	-	-
RPL22	0.735	0.671	-	-	-	-	-
p-value	0.001	0.004	-	-	-	-	-
YWHAZ	0.707	-0.384	0.300	-	-	-	-
p-value	0.002	0.142	0.259	-	-	-	-
EEF1A1	0.718	0.705	0.908	0.150	-	-	-
p-value	0.002	0.002	0.001	0.580	-	-	-
RPL0	0.183	0.853	0.643	-0.343	0.705	-	-
p-value	0.499	0.001	0.007	0.194	0.002	-	-
RNA18SN5	0.414	0.309	0.571	0.156	0.548	0.360	-
p-value	0.111	0.245	0.021	0.563	0.028	0.170	-

Pearson correlation coefficient (r)

BestKeeper vs.	PPIB	GAPDH	RPL22	YWHAZ	EEF1A1	RPL0	RNA18SN5
coeff. of corr. [r]	0.612	0.555	0.822	0.204	0.806	0.583	0.923
p-value	0.012	0.026	0.001	0.448	0.001	0.018	0.001

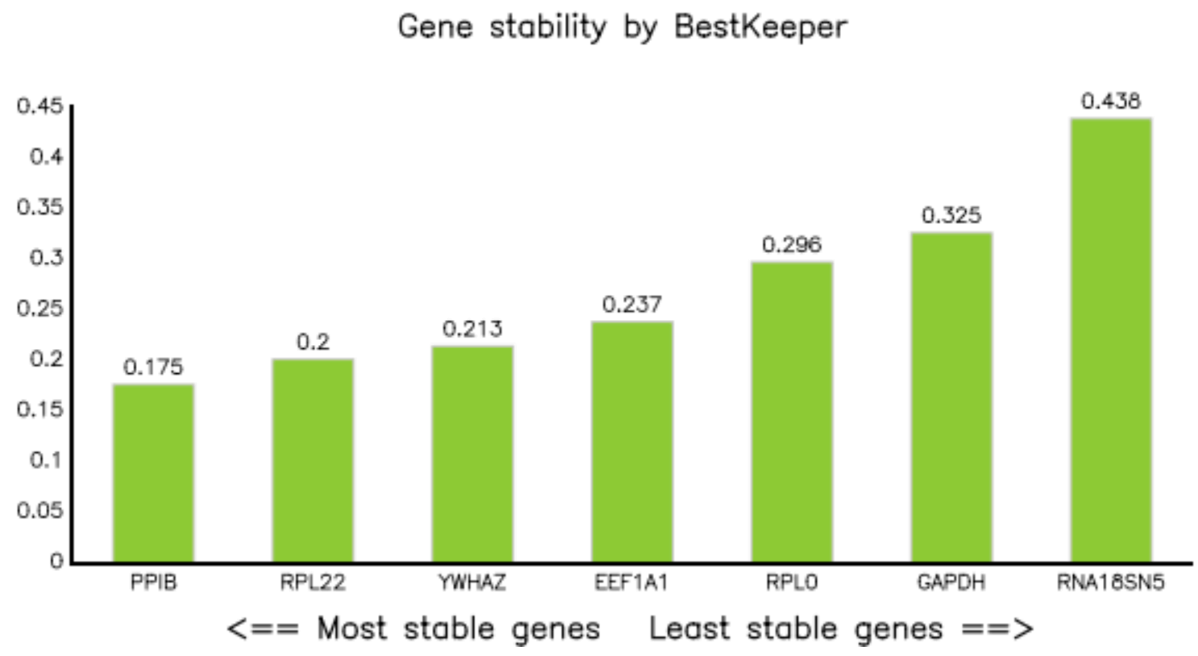

Supplement 2: RefFinder results

normFinder

Gene name	Stability value
EEF1A1	0.007
RPL22	0.063
PPIB	0.197
RPL0	0.259
GAPDH	0.297
YWHAZ	0.346
RNA18SN5	0.642

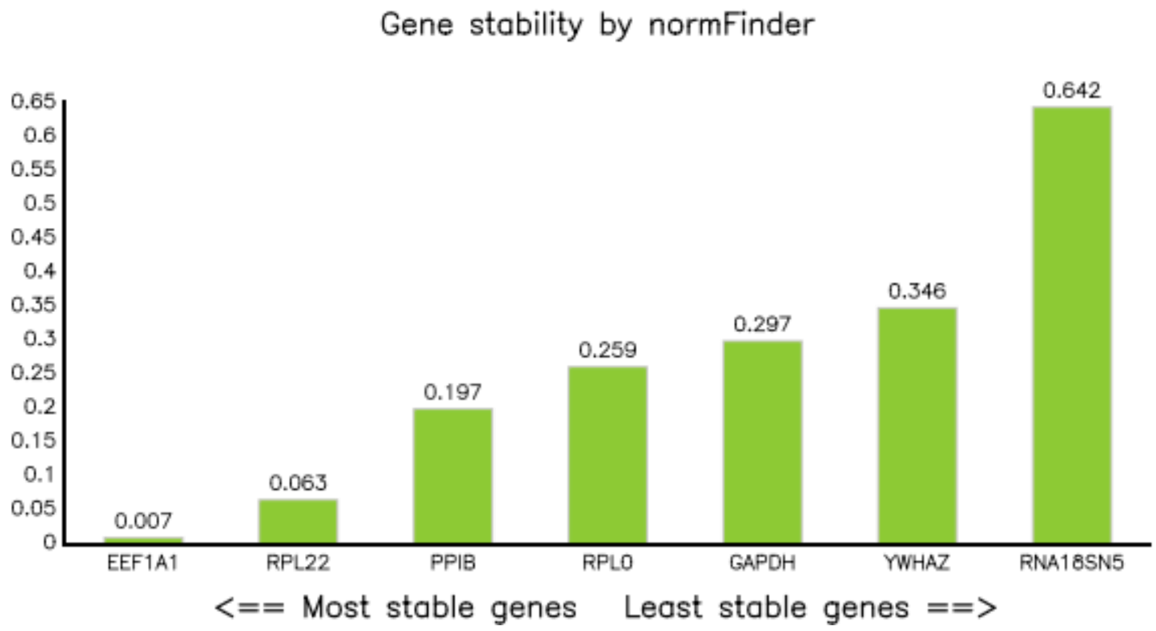

Supplement 2: RefFinder results

Genorm

Gene name	Stability value
RPL22 | EEF1A1	0.126
RPL0	0.217
GAPDH	0.233
PPIB	0.263
YWHAZ	0.300
RNA18SN5	0.408

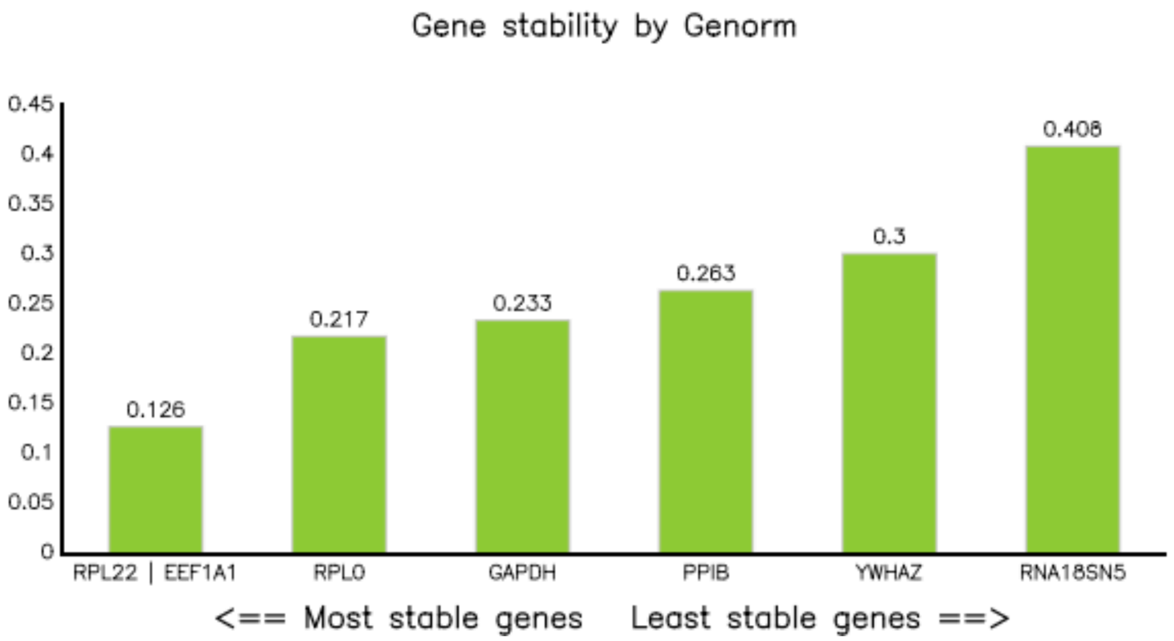

Supplement: Supplementary file 1 [file ijms-25-13513-s001.zip › Supplement_File S2_SH009_RefFinder Results_FINAL.pdf]
